# Supplementary material for: Surgeon interpretation of patient-reported outcome measures in upper extremity osteoarthritis
Source: PLoS One. 2025 Sep 12;20(9):e0332221. doi: 10.1371/journal.pone.0332221 (PMC12431210; doi:10.1371/journal.pone.0332221)
Supplement: S1 Appendix — (DOCX) [file pone.0332221.s001.docx]

**Appendix A: Preparatory Experiments**

Surgeons of the Science of Variation Group (SOVG) completed two preliminary experiments using structured questionnaires that presented specific patient scenarios with scores on patient reported outcome measures and mental health measures.

**Experiment 1**

In the first experiment, we explored 156 surgeons' practices regarding patient-reported measures quantifying capability and mental health, more specifically: how often they review them when caring for individual patients, which measures they use, how patient-reported measures may affect practice, interpretation of scores (threshold scores in particular), and whether there were any patient characteristics that would influence the surgeon's interpretation of these scores. Open text responses were analyzed using an inductive coding method, specifically thematic analysis^1^. If two independent observers were unable to understand responses or when the responses were not related to the question, they were coded as ambiguous and omitted. Most participants were men (88%), practiced in the United States (47%), and supervised trainees (83%). Among surgeons who routinely collected patient-reported measure or mental health measures, the most commonly used tools for assessing capability were Quick Disabilities of the Arm, Shoulder, and Hand (Quick-DASH) (35%), Patient-Reported Outcomes Measurement Information System (PROMIS) Upper Extremity Physical Function Computerized Adaptive Test (CAT): (23%), EuroQol-5 Dimensions (EQ-5D): (18%), Visual Analog Scale (VAS) pain score: (11%), Patient-Reported Outcomes Measurement Information System (PROMIS) Depression CAT and Anxiety CATs (together accounting for 38%) emerged as the predominant tool for mental health measurement. The vast majority of the participants 126 of 138 (91%) did not utilize thresholds for patient-reported measure scores to decide on tests or treatments. Among those who did, cutoff points were specific to each patient-reported measure. For example, “Simple Shoulder Test must be lower than 7 to offer (reversed) total shoulder arthroplasty”, these cutoff points could motivate tests or other treatment strategies. Most participants, 129 of 137 (94%), did not use threshold scores on mental health measurements as triggers for alternative treatment choices such as prioritizing mental health over tests and treatments addressing pathophysiology. For the minority who did, specific cutoff points on mental health measures, such as “60 for PROMIS Anxiety/Depression” or “9-item Patient Health Questionnaire score > 18” these scores were used to trigger management that prioritized mental health.

| **Collection of patient-reported measures quantifying capability and mental health.** |
| --- |
| Among the 40% (62 of 156) of surgeons that collect patient-reported measure scores |
| a. 45% (28 of 62) review them before the visit |
| b 27% (17 of 62) review them during the visit |
| c. 56% (35 of 62) review them after the visit |
| Among the 14% (21 of 148) of surgeons that collect mental health measures |
| a. 57% (12 of 21) Review them before the visit |
| b. 33% (7 of 21) Review them during the visit |
| c. 57% (12 of 21) Review them after the visit  *Surgeons could select more than one time point. |

| **Alteration of care strategies based on patient-reported measures quantifying capability or mental health.** | |
| --- | --- |
| 53 Participants who altered care strategies based on the scores of patient-reported measures. | |
| *Themes identified among open-text response* |  |
| To gain an understanding of patient perspective |  |
| To use in decision making |  |
| To track patient progress |  |
| To understand patient mental health |  |
| For shared decision making |  |
| No alteration in practice |  |

| **Treatment strategies when patient has unexpectedly high discomfort and incapability.** | |
| --- | --- |
| *Themes identified among open-text response* |  |
| Alter treatment recommendation |  |
| Consider mental health issues |  |
| Speak with the patient |  |
| Seek another pathophysiology |  |
| Lower their appeal for surgery |  |
| Offer pain treatment or examine current treatment |  |
| Document |  |
| Have multi/interdisciplinary consultation |  |
| Have a higher appeal for surgery |  |

| **Patient characteristics that make surgeons pay more attention to patient-reported measures quantifying capability or mental health.** | |
| --- | --- |
| *Themes identified among open-text response* |  |
| No specific characteristics |  |
| Mental health diagnosis or symptoms |  |
| Out of proportion or diffuse symptoms |  |
| Secondary gain (lawsuit, work compensation) |  |
| Demeanor |  |
| Pain medication or substance abuse |  |

**Experiment 2**

In the second experiment, we presented four fictional patient scenarios of musculoskeletal illness to 76 participants with similar demographics as in experiment 1. Among the four fictional patient scenarios, two addressed patient-reported measures quantifying capability and two addressed patient-reported measures quantifying mental health: one each with a Gaussian and a non-Gaussian score distribution. Since we simulated a new patient specialty visit, no specific diagnosis or clinical details were included in the scenarios, nor were thresholds for change in scores over time, such as minimal clinically important difference values. Surgeons were asked to identify scores that concerned them, explain the reasons for their concern, and comment on patient characteristics that might influence their interpretations.

*Capability scenarios*

In the first scenario, participants were presented the description of a capability measure with a Gaussian distribution, where the scores ranged from 0 to 100 with 50 resembling the mean of a population with an unspecified longstanding musculoskeletal condition (no specific conditions were mentioned as we wanted to keep the cases broad and relevant for every subspecialty) and every 10 points above or below a standard deviation. Higher scores indicated worse capability. Surgeons were concerned, on average, with scores that were 2 standard deviations above the mean (2.5% of the population).
In the second scenario, participants were presented the description of a capability measure with a non-Gaussian distribution, where the scores ranged from 0 to 100 with 31 resembling the median of a population with an unspecified longstanding musculoskeletal condition with an interquartile range of 23 to 52. Higher scores indicated worse capability. Surgeons were concerned, on average, with patients in the top most incapable quartile (25% of the population).

*Mental health scenarios*

In the third scenario, participants were presented the description of a mental health measures with a Gaussian distribution, where scores ranged from 0 to 100 with 50 resembling the mean of a population with an unspecified longstanding musculoskeletal condition and every 10 points above or below a standard deviation. Higher scores indicated worse mental health. Surgeons were concerned, on average, with scores of mental health measurement that were 1.5 standard deviations worse than the mean (6.5% of the population).

In the fourth scenario, participants were presented the description of a mental health measure with a non-Gaussian distribution, where scores ranged from 3 to 15, with 6 resembling the median of a population with an unspecified longstanding musculoskeletal condition with an interquartile range of 4 to 9. Higher scores indicated worse mental health Surgeons were concerned, on average, with scores in the quartile with the worst mental health scores (25% of the population).

*Interobserver agreement*

We then proceeded to assess the interobserver agreement of the threshold score of concern among participants using a two-way mixed effect intraclass correlation analysis (ICC). Only the scenarios with scores ranging between 0 and 100 were included as the ICC model requires stable variance, which was not the case for the mental health scenario with non-Gaussian distribution (scores ranging between 3 and 15). The interobserver reliability was 0.21 (95% CI: 0.054 to 0.92), representing poor agreement according to Shrout and Fleiss^2^. The low interobserver agreement score of 0.21 suggests significant variability in how surgeons interpreted threshold scores of concern. This raises the question of whether there is a lack of consensus among participants. The wide range of scores assigned indicates that surgeons may not have uniformly interpreted the cases. The unfamiliarity of the surgeons with the patient-reported outcome measures (PROMs) could have influenced their level of agreement.

**Results of open-text answers to capability scenarios**

| **Rationale for the specific given scores by the surgeons in capability cases.** | |
| --- | --- |
| *Themes* | *Quotes from the responses* |
| Outlier | "Roughly more than 2SD from the median" |
| Possible mental health issues | "Suggests relationship between physical function and mental health" |
| Diagnosis may be altered/other factors at play | "There are other factors beyond the MSK issues affecting the score" |
| Patient compliance/Ability of patient to fill in patient-reported measures | "I would question diagnosis or patients’ honesty in completing PROM" |
| Lower appeal for surgery | "Other interventions than surgical procedures maybe more effective" |

| **Actions surgeons would take on concerning scores in capability cases.** | | | |
| --- | --- | --- | --- |
| *Themes* |  | | *Quotes from the responses* |
| Speak with patient |  | "Discuss this with the patient" | |
| Seek pathophysiology |  | "Detailed history physical and investigations" | |
| Alter treatment |  | "It would affect my treatment and recommendations" | |
| Think about psychosocial factors and psychosocial referral |  | "Refer to Think about psychosocial factors and psychosocial referral provider" | |
| Lower appeal for surgery |  | "Consider additional consultation and less likely to recommend surgery" | |
| Higher appeal of surgery |  | "Surgery" | |

| **Barriers that surgeons feel that prevent actions in capability cases.** | | |
| --- | --- | --- |
| *Themes* |  | *Quotes from the responses* |
| Patient Factors (e.g. trust, adherence) |  | "Patient assumptions on surgical cures" |
| Psychosocial factors (including secondary gain) |  | "Personal beliefs in general, family matters, workers’ compensation" |
| No barriers |  | "I cannot think of any such barriers" |
| Access to appropriate referral |  | "Tertiary care means no referral elsewhere" |
| Time |  | "Nothing just time pressure" |
| Surgeon understanding of scores, communication strategies, and resources |  | "Uncomfortable speaking about the elephant in the room" |

| **Additional information surgeons would like to know in capability cases.** | | |
| --- | --- | --- |
| *Themes* |  | *Quotes from the responses* |
| Psychosocial status |  | "Information about the psychosocial context" |
| Medical history |  | "Complete (medical) history" |
| Does not need extra information |  | "No" |
| Seek pathophysiology |  | "Physical exam, special tests" |
| Medication |  | "Drugs and medication" |

| **Patient characteristics that would change surgeon interpretation of scores in capability cases.** | | |
| --- | --- | --- |
| *Themes* |  | *Quotes from the responses* |
| Psychosocial (including secondary gain) |  | "Catastrophic thinkers, lawsuit, chronic pain patients" |
| No characteristics |  | "None" |
| Medical history |  | "Prior surgery" |
| Pathophysiology |  | "Yes chronic pain, disability" |
| Substance abuse |  | "Substance abuse " |
| Interaction with patient |  | "Discussion with patient" |
| Ability of patient to fill in the patient-reported measures / information about patient reported measures and previous scores | | "Lack of understanding of the PROM" |

| **Patient characteristics that would change surgeon interpretation of scores in capability cases when the patient has injury instead of a longstanding disease^a^** | |
| --- | --- |
| *Themes* | *Quotes from the responses* |
| Psychosocial (including secondary gain) | "Litigation " |
| No characteristics | "None" |
| Pathophysiology | "Yes, type of injury and severity" |
| Trauma mechanism/baseline pre-injury | "Would compare with pre-injury baseline" |
| Substance abuse | "Chronic opioid abuse" |
| Medical history | "History and drugs" |
| ^a^Question was only asked in scenario 2. | |

**Results of open-text answers to mental health scenarios**

| **Rationale for the specific given scores by the surgeons in mental health cases.** | |
| --- | --- |
| *Themes* | *Quotes from the responses* |
| Outlier | "Above 2SD" |
| Possible mental health issues | "Likelihood of abnormal illness behavior" |
| Concern about effectiveness/complications with interventions | "More likely to develop post-op pain and/or complications" |

| **Actions surgeons would take on concerning scores in mental health cases.** | |
| --- | --- |
| *Themes* | *Quotes from the responses* |
| Speak with patient | "Asking the patient first" |
| Think about psychosocial factors and psychosocial referral | "Consider to refer to psychologist" |
| Lowers appeal for surgery | "Reticence to recommend surgery" |
| Seek pathophysiology | "Exam, questioning and imaging" |
| Contact with primary MD/Multidisciplinary meeting | "Follow up with PCP " |

| **Barriers that surgeons feel that prevent actions in mental health cases** | |
| --- | --- |
| *Themes* | *Quotes from the responses* |
| Patient Factors (e.g. trust, adherence) | "Patient skepticism/expectations of care" |
| Psychosocial factors (including secondary gain) | "Social determinants of health" |
| Access to appropriate referral | "Lack of access to mental healthcare" |
| No barriers | "None" |
| Time | "Time constraints to go into details" |
| Surgeon understanding of scores, communication strategies, and resources | "Not trained as mental health specialist" |

| **Additional information surgeons would like to know in mental health cases** | |
| --- | --- |
| *Themes* | *Quotes from the responses* |
| Psychosocial status | "Psychological support of the patient" |
| Medical history | "History and physical, mental health history" |
| Does not need extra information | "No" |
| Seek pathophysiology | "Clinical examination" |

| **Patient characteristics that would change surgeon interpretation of scores in mental health cases** | |
| --- | --- |
| *Themes* | *Quotes from the responses* |
| No characteristics | "None" |
| Psychosocial (including secondary gain) | "Psychosocial issues" |
| Medical history | "Known psychiatric diagnosis" |

| **Patient characteristics that would change surgeon interpretation of scores in mental health cases when the patient has injury instead of a longstanding disease^a^** | |
| --- | --- |
| *Themes* | *Quotes from the responses* |
| Psychosocial status (including secondary gain) | "Social determinant of health including insurance status" |
| No characteristics | "No" |
| Trauma mechanism/baseline pre-injury | "Mechanism of injury" |
| Medical history | "History" |
| ^a^Question was only asked in scenario 4. | |

**Reference**

1. Thomas, J, Harden, A: Methods for the thematic synthesis of qualitative research in systematic reviews. *BMC Med Res Methodol* 2008;8:

2. Shrout, PE, Fleiss, JL: Intraclass correlations: uses in assessing rater reliability. *Psychol Bull* 1979;86:420–428.
