# Supplementary material for: Surgeon interpretation of patient-reported outcome measures in upper extremity osteoarthritis
Source: PLoS One. 2025 Sep 12;20(9):e0332221. doi: 10.1371/journal.pone.0332221 (PMC12431210; doi:10.1371/journal.pone.0332221)
Supplement: S2 Appendix — (DOCX) [file pone.0332221.s002.docx]

**Appendix B**

*Survey corresponding to Appendix A experiments:*

1. Do you collect PROMs routinely? (y/n)
2. What PROMs do you use in your clinical practice?
3. If so, when do you usually look at PROMs when you see the patient?
   1. before the visit? (y/n)
   2. during the visit? (y/n)
   3. after the visit? (y/n)
4. Do you collect mental health measures routinely?
5. What mental health measures do you use in your clinical practice?
6. When do you usually look at mental health measures when you see the patient?
   1. before the visit? (y/n)
   2. during the visit? (y/n)
   3. after the visit? (y/n)
7. Does having PROMs or mental health measures available alter your practice? (y/n)
   1. Please explain (text box)
8. Are there thresholds in PROM scores you use to decide on tests and treatments? (y/n)
   1. Please explain (text box)
9. Are there thresholds of mental health scores you use to decide on tests and treatments? (y/n)
   1. Please explain (text box)
10. When a patient has greater discomfort and incapability than you expect, how do you use that information? (text box)
11. Are there any patient characteristics that make you pay more attention to PROM or mental health scores? (text box)

A patient with a longstanding musculoskeletal condition presents to specialty care for the first time. You use a PROM that ranges in score from 0 (full capability) to 100 (complete incapability). Roughly 50% of the patients seen with this MSK condition score between 23 and 52, and the median is 31.

1. Above what score would you be concerned? (number)
2. Why would that score concern you? (text box)
3. How would you act on the concerning score? (text box)
4. What barriers do you feel that might prevent specific possible actions? (text box)
5. Is there any other information you would like to know? (text box)
6. Are there any patient characteristics that would change your interpretation of the score? (text box)

A patient with a longstanding musculoskeletal condition presents to specialty care for the first time. You use a PROM that ranges in score from 0 (full capability) to 100 (complete incapability). The mean score for patients with this MSK condition is 50 and each 10 points above or below represents 1 standard deviation.

1. Above what score would you be concerned? (number)
2. Why would that score concern you? (text box)
3. How would you act on the concerning score? (text box)
4. What barriers do you feel that might prevent specific possible actions? (textbox)
5. Is there any other information you would like to know? (text box)
6. Are there any patient characteristics that would change your interpretation of the score? (text box)
7. Now imagine that this patient had an injury rather than longstanding disease such as arthritis or tendinopathy. Are there any patient characteristics that would change your interpretation of the scores? (text box)

A patient with a longstanding musculoskeletal condition presents to specialty care for the first time. You use a mental health score in your clinic that ranges in score from 0 (best) to 100 (worst). The mean score for patients with this condition is 50 and each 10 points above or below represents 1 standard deviation.

1. Above what score would you be concerned? (number)
2. Why would that score concern you? (text box)
3. How would you act on the concerning score? (text box)
4. What barriers do you feel that might prevent specific possible actions? (text box)
5. Is there any other information you would like to know? (text box)
6. Are there any patient characteristics that would change your interpretation of the score? (text box)

A patient with a longstanding musculoskeletal condition presents to specialty care for the first time. You use a mental health score in your clinic that ranges in score from 3 (best) to 15 (worst). Roughly 50% of patients score in between 4 and 9, with median of 6, and 25% of the patients scores the lowest score of 3.

1. Above what score would you be concerned? (number)
2. Why would that score concern you? (text box)
3. How would you act on the concerning score? (text box)
4. What barriers do you feel that might prevent specific possible actions? (text box)
5. Is there any other information you would like to know? (text box)
6. Are there any patient characteristics that would change your interpretation of the score? (text box)
7. Now imagine that this patient had an injury rather than longstanding disease such as arthritis or tendinopathy. Are there any patient characteristics that would change your interpretation of the scores? (text box)

*Survey corresponding to main experiment*

*general information provided with each scenario:*

PLEASE NOTE: In all cases the scores change, but also the ranges and information about PROM score. Please read them carefully.

The scores are all scaled so that 50 is the general ​​population mean and 10 points higher (worse) or lower (better) represents a standard deviation.

For capability, levels of distress (symptoms of distress and unhelpful thinking) and multi deprivation index all score can be interpretated the same way. A score of 100 is worst possible score and 0 is best score possible.  Pain intensity score is presented on a scale from 0 (no pain) to 10 (worst pain imaginable). The MDI index is based on: standard of living, health, education, economic security, housing quality, and neighborhood quality.


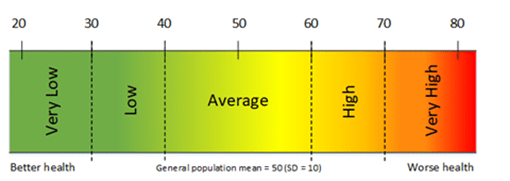
 
The figure above was shown to the participants during the survey.

*Scenarios:*

A {45/ 61/ 83} year-old {random 50% man; 50% woman} comes to your office for an initial specialty care visit.  The diagnosis is {TMC/SLAC/GH OA} with radiographic severity of ​​{TMC: grade 1 / 2 / 3 & wrist/elbow: grade 2 / 3 / 4} and the level of distress and unhelpful thinking is [34 / 52 / 73}”. They live in an area with a level of deprivation index of {25 / 54 / 79}. The pain intensity and the level of incapability are {9 and 71 ; 5 and 49 ;  2 and 30} ​​respectively.
